# Supplementary material for: Silicosis, tuberculosis and silica exposure among artisanal and small-scale miners: A systematic review and modelling paper
Source: PLOS Glob Public Health. 2023 Sep 21;3(9):e0002085. doi: 10.1371/journal.pgph.0002085 (PMC10513209; doi:10.1371/journal.pgph.0002085)
Supplement: S1 Table — (DOCX) [file pgph.0002085.s008.docx]

# S1 Table. Characteristics of studies reporting respiratory symptoms (n=3) and smoking (n=8) or substance misuse (n=2) estimates among ASM

| Author, year | Study country, period | Study design | Population | Sample size | Sampling method | Age (years), gender | Symptoms ASM | Symptoms Controls | Smoking or substance misuse prevalence |
| --- | --- | --- | --- | --- | --- | --- | --- | --- | --- |
| Symptoms | | | | | | | | | |
| Leon-Kabamba, 2018(1) | Democratic Republic of Congo, 2016 | Exposure-control | Above ground coltan miners and government office workers | 441 (199 ASM, 242 community controls) | 199/247 miners from single mine, controls from local administrative building | ASM: 32.8 +/- SD 8.3 years  Controls: 33.9 +/- 9.3 years  No gender data | Shortness of breath: 55/199 (27.6%)  Morning cough: 95/199 (47.7%)  Wheeze at rest: 85/199 (42.7%) | Shortness of breath: 6/242 (2.5%)  Morning cough: 6/242 (2.5%)  Wheeze at rest: 6/242 (2.5%) | ASM: 115/199 (58%)  Controls: 22/242 (9.1%) |
| Ralph, 2018(2) | Cameroon, 2018 | Cross-sectional | Current or retired above and underground gold ASM | 273 (174 ASM and 99 controls) | Mix of stratified random and convenience sampling | ASM: mode group 26-35 years (37.9%), 132/174 (75.9%) male  Controls: mode group 36-50 years (42.4%), 57/99 (57.6%) male | Breathlessness: 6/174 (3.6%)  Persistent cough: 54/174 (31.0%) | Breathlessness: 0/99 (0%)  Persistent cough: 9/99 (9.1%) | ASM: 72/174 (41.4%) |
| Souza, 2021(3) | Brazil, 2017-2018 | Cross-sectional | Current underground precious stone ASM | 258 | Miners from 49/277 randomly selected mines | Mean 40 (SD +/- 16.0) years, all male | Dyspnoea: 29/258 (11.2%)  Cough: 65/258 (25.2%) | - | 46/258 (17.8%) |
| Smoking | | | | | | | | | |
| Osim, 1999(4) | Zimbabwe, no date described | Exposure-control | Above and below ground chrome ASM, chrome LSM and community controls | 54 ASM , 46 LSM and 50 community controls | Not described | ASM: 32.7 +/- 1.5  LSM: 33.3 +/- 1.2  Controls: 31.6 +/-1.5,  All male | - | - | ASM: 9/54 (16%)  LSM: 7/50 (14%)  Controls: 7/50 (15%) |
| Tse, 2007(5) | China, 1997-2001 | Cross-sectional | Retired underground gold ASM rock-drillers | 583 | Complete sample of rock-drillers | 24.4 +/- SD 6.7 years, All male | - | - | 115/583 (35.1%) |
| Souza, 2017(6) | Brazil, 2013-2014 | Cross-sectional | Current and retired underground precious stone ASM | 348 | Sequential sample of registered miners attending annual screening | Mean 40.1 (SD +/- 11.9) years, all male | - | - | 81/348 (23.3%) |
| Kyaw, 2020(7) | Myanmar, 2020 | Cross-sectional | ASM gold miners and local community controls | 18 ASM, 11 controls | Randomly recruited from town; no specified method | ASM: 37.6 +/- 15.2, 66% male  Controls: 56.1 +/- 13.9, 55% male | - | - | ASM: 8/18 (44%)  Controls: 2/11 (18%) |
| Mbuya, 2023(8) | Tanzania, 2019-2021 | Cross-sectional | Below ground gemstone ASM | 330 | 15 randomly sampled miners from 22 randomly chosen mines | Median 35.0 (IQR 30.0–44.0),  All male |  |  | 60/330 (18.2%) |
| Substance misuse | | | | | | | | | |
| Moyo, 2021(9) | Zimbabwe, 2020-2021 | Cross-sectional | Current artisanal gold miners | 514 | Miners attending TB outreach screening and hospital occupational health clinic | Mean 37.0 (SD +/- 12.7) years, 435/514 (85%) male | - | - | Marijuana use: 143/365 (28%) |
| Moyo, 2022(10) | Zimbabwe, 2020-2022 | Cross-sectional | Above or underground gold and chrome ASM | 3950 | Miners attending TB outreach screening and hospital occupational health clinic | Mean 35.5 (SD +/- 12.1) years, 3245/3950 (85%) male | - | - | "Substance use" 673/3098 (17.0%) |

References:

1. Leon-Kabamba N, Ngatu NR, Kakoma SJB, Nyembo C, Mbelambela EP, Moribe RJ, et al. Respiratory health of dust-exposed Congolese coltan miners. Int Arch Occup Environ Health. 2018;91(7):859–64.

2. Ralph O, Gilles N, Fon N, Luma H, Greg N. Impact of artisanal gold mining on human health and the environment in the Batouri Gold District, East Cameroon. Acad J Interdiscip Stud. 2018;7(1):25–44.

3. Souza TP, Tongeren M, Monteiro I. Respiratory health and silicosis in artisanal mine workers in southern Brazil. Am J Ind Med. 2021 Jun;64(6):511–8.

4. Osim EE, Tandayi M, Chinyanga HM, Matarira HT, Mudambo KK, Musabayane CT. Lung function, blood gases, pH and serum electrolytes of small-scale miners exposed to chrome ore dust on the Great Dyke in Zimbabwe. Trop Med Int Health TM IH. 1999;4(9):621–8.

5. Tse LA, Li ZM, Wong TW, Fu ZM, Yu ITS. High prevalence of accelerated silicosis among gold miners in Jiangxi, China. Am J Ind Med. 2007;50(12):876–80.

6. Souza TP, Watte G, Gusso AM, Souza R, Moreira J da S, Knorst MM. Silicosis prevalence and risk factors in semi-precious stone mining in Brazil. Am J Ind Med. 2017 Jun;60(6):529–36.

7. Kyaw WT, Kuang X, Sakakibara M. Health Impact Assessment of Artisanal and Small-Scale Gold Mining Area in Myanmar, Mandalay Region: Preliminary Research. Int J Environ Res Public Health. 2020;17(18).

8. Mbuya AW, Mboya IB, Semvua HH, Mamuya SH, Msuya SE. Prevalence and factors associated with tuberculosis among the mining communities in Mererani, Tanzania. Tsima BM, editor. PLOS ONE. 2023 Mar 15;18(3):e0280396.

9. Moyo D, Zishiri C, Ncube R, Madziva G, Sandy C, Mhene R, et al. Tuberculosis and Silicosis Burden in Artisanal and Small-Scale Gold Miners in a Large Occupational Health Outreach Programme in Zimbabwe. Int J Environ Res Public Health. 2021;18(21).

10. Moyo D, Ncube R, Kavenga F, Chikwava L, Mapuranga T, Chiboyiwa N, et al. The Triple Burden of Tuberculosis, Human Immunodeficiency Virus and Silicosis among Artisanal and Small-Scale Miners in Zimbabwe. Int J Environ Res Public Health [Internet]. 2022;19(21).
