# Supplementary material for: Silicosis, tuberculosis and silica exposure among artisanal and small-scale miners: A systematic review and modelling paper
Source: PLOS Glob Public Health. 2023 Sep 21;3(9):e0002085. doi: 10.1371/journal.pgph.0002085 (PMC10513209; doi:10.1371/journal.pgph.0002085)
Supplement: S2 Table — (DOCX) [file pgph.0002085.s009.docx]

# S2. Table Characteristics of studies reporting spirometry (n=3) estimates among ASM

| Author, year | Study country, period | Study design | Population | Sample size | Sampling method | Spirometry method | Age (years), gender | FEV1 | FVC | FEV1/FVC (%) |
| --- | --- | --- | --- | --- | --- | --- | --- | --- | --- | --- |
| Osim, 1999(1)* | Zimbabwe, no date described | Exposure-control | Above and below ground chrome ASM, chrome LSM and community controls | 54 ASM , 46 LSM and 50 community controls | Not described | Three attempts at spirometry were allowed. Measurements required to meet ATS criteria (1979) | ASM: 32.7 +/- 1.5  LSM: 33.3 +/- 1.2  Controls: 31.6 +/-1.5,  All male | ASM: 2.6L +/- 0.1L  LSM: 3.1L +/- 0.1L  Controls: 3.2L +/- 0.1L | ASM: 3.5L +/- 0.1L  LSM: 3.9L +/- 0.1L  Controls: 3.2L +/- 0.1L | ASM: 76.2% +/- 2.4%  LSM: 81.8% +/- 1.4%  Controls: 80.7% +/- 1.5% |
| Rajaee, 2017(2) | Ghana, 2011 | Cross-sectional | ASM current and ex-gold miners and local community controls | 57 ever miners, 14 never miners | Random sampling within stratified geographic clusters for Kejetia | Comprehensive and standardised methodology described. Excluded if criteria not met (159/172 of overall sample) | Not described | ASM: Male 88.7% (SD +/- 11.1). Female 87.4% (SD +/- 14.0)  Controls: Male 93.8% (SD +/- 20.2)  Female 88.8% (SD +/- 9.4) | ASM: Male 92.7% (SD +/- 10.0). Female 92.0% (SD +/- 11.2). Controls: Male 92.7% (SD +/- 20.7). Female 94.3% (SD +/- 10.8) | ASM: Male 92.9% (SD +/- 9.0). Female 95.1% (SD +/- 10.9)  Control: Male 102.1% (SD +/- 3.8)  Female 91.0% (SD +/- 4.5) |
| Kyaw, 2020(3) | Myanmar, 2020 | Cross-sectional | ASM underground gold miners and local community controls | 18 ASM, 11 controls | Randomly recruited from town; no specified method | Three attempts with best result used. If not successful repeated until success | ASM: 37.6 +/- 15.2, 66% male  Controls: 56.1 +/- 13.9, 55% male | ASM: 2.49L (IQR 2.06-3.35L),  81.5% (IQR 71.3–90.8%)  Control: 2.01L (IQR 1.71-2.51L),  83.0% (IQR 64.5–91.5%) | ASM: 2.89L (IQR 2.26-3.37L),  75.5% (IQR 64.5–86.8%)  Controls: 2.11L (1.77–2.59L), 77.0% (IQR 55.5–80.0%) | - |

* Similar smoking prevalence; ASM 9/54 (16%), LSM 7/46 (15%) and controls 7/50 (15%).

# ASM: 8/18 (44%) smokers, controls: 2/11 (18%) smokers. Significant methodological issues with study

References:

1. Osim EE, Tandayi M, Chinyanga HM, Matarira HT, Mudambo KK, Musabayane CT. Lung function, blood gases, pH and serum electrolytes of small-scale miners exposed to chrome ore dust on the Great Dyke in Zimbabwe. Trop Med Int Health TM IH. 1999;4(9):621–8.

2. Rajaee M, Yee AK, Long RN, Renne EP, Robins TG, Basu N. Pulmonary function and respiratory health of rural farmers and artisanal and small scale gold miners in Ghana. Environ Res. 2017;158(ei2, 0147621):522–30.

3. Kyaw WT, Kuang X, Sakakibara M. Health Impact Assessment of Artisanal and Small-Scale Gold Mining Area in Myanmar, Mandalay Region: Preliminary Research. Int J Environ Res Public Health. 2020;17(18).
