# Supplementary material for: Silicosis, tuberculosis and silica exposure among artisanal and small-scale miners: A systematic review and modelling paper
Source: PLOS Glob Public Health. 2023 Sep 21;3(9):e0002085. doi: 10.1371/journal.pgph.0002085 (PMC10513209; doi:10.1371/journal.pgph.0002085)
Supplement: Attachment — Submitted filename: Response to Reviewers.docx [file pgph.0002085.s011.docx]

**Reviewer comments**

Reviewer #1: The study by Howlett et al highlights a major public health topic on silicosis, TB in ASM. The paper is well written and has great merit and is likely to gain much traction during the period leading to the UN-HLM in September.

On behalf of the study authors; thank you. We believe this is an important topic and hope that this scientific article can be used to inform policy debate in September.

The authors may highlight that there is need for multisectoral approaches involving Ministries of mines and Health-NTPs and Environmental Health specialties in order to implement the recommendations they suggested. Finally, given that ASM are hypermobile and usually operate in hard to reach and underserved areas, TB services (diagnostic and case holding) need to be tailored to the specific needs of this group.

Thank you for this comment which is also raised by reviewer 2 (Discussion 1). We have included 2 sentences in line 493-497, that references the paper by Rupani and includes the helpful comments above:

*“There exists consensus among experts that TB control in mining populations requires a multisectoral approach(66). The challenging of doing this however should not be underestimated; SSM populations are often hypermobile and operate in hard-to-reach and historically underserved areas.”*

Reviewer #2: General
1. Comments in abstract should also be addressed in the full text, where applicable.

Title and abstract
1. The authors can remove the colon in the title and keep a continuous line.

If the editor and reviewer allow, we would kindly request to keep the colon as this is the authors preference. However, if we are requested to change the title, it may be changed to *“A systematic review and modelling study of silicosis, tuberculosis and silica exposure among artisanal and small-scale miners”.*

2. What was the research question behind this systematic review? As such, I could not pin point anything novel in the research question/ objective. Please describe the novelty which the authors targeted.

Many thanks for this comment, we have tried to clarify the novelty and hypothesis of our study. In our abstract we have therefore clarified (L51-53) that:

*“An estimated 44 million artisanal and small-scale miners (ASM), largely based in developing economies, face significant occupational risks for respiratory diseases which have not been reviewed.* *We therefore aimed to review studies…”*

In our main text (L128-132), we have redrafted to:

*“More precisely, we hypothesise that, among ASM, high RCS exposures lead to a high prevalence of silicosis and tuberculosis which is associated with abnormal spirometry and a high respiratory symptom burden. Higher background TB and HIV rates compound these exposure risks. We are not aware of a previous systematic review that investigates this hypothesis.”*

3. The relationship between silica dust exposure, silicosis and tuberculosis is very well known (<https://bmcpublichealth.biomedcentral.com/articles/10.1186/s12889-021-10711-1>). What did the authors add to the existing body of evidence?

Many thanks for this comment. We agree that the relationship is very well established. We are not introducing any new primary data to this relationship. Instead, we are using utilising existing evidence and combining this with our causal understanding of the problem to observe and extrapolate how silicosis and TB disease risks may change with respect to cumulative silica exposure, particularly at higher exposures seen among ASM. Put more straightforwardly, this may be considered as ‘drawing our assumptions’(1). As such, we do not propose that the values produced in the models are exact, although they may provide helpful context for those of the systematic review. Instead, our main aim was in being able to observe different scenarios (counter-factual scenarios) to improve our understanding of what is happening “in the real world”. We believe these models are helpful and novel as no studies from ASM populations (or similar high exposure populations) link either individual or mine silica exposures to respiratory outcomes; models may therefore represent our prior estimates and assumptions. Furthermore, there are no studies which adjust for all of silica exposure, silicosis and HIV despite, as outlined in Fig 1. It is our belief that there is good evidence and conceptual support for these variables all to form part of the causal relationship between silica exposure and TB risk in miners (as outlined in Supplementary File 6).

To try and reflect the above, we have amended our introduction to state (L134-147):

*“Observational studies linking individual exposures, silicosis risk and TB incidence among LSM are comprehensive and generally good quality(16,17), although none adjust for both RCS and HIV as implied by our conceptual framework (Fig 1.). We are not aware of similar observational data for ASM, in whom silica exposure may be higher, and no interventional studies linking dust reduction to respiratory outcomes in any setting have been reported. In the absence of such data, extrapolating from LSM studies and using our conceptual framework (Fig. 1) may improve our understanding of the determinants of silicosis and TB.”*

4. Define ASM in the abstract itself.

Thank you also for this comment. To the best of our knowledge there is no standardised or widespread definition of ASM in use. Indeed, the most widely referenced estimates of ASM populations (the DELVE publications) do not provide the definitions used for their estimates. A recent scoping review(2) starts by defining ASM as: “commonly defined as any mining activity of ore deposits that are considered not cost-effective for large-scale industrial extraction”. We are not aware, however, that this is a commonly used definition. The article also references a set of shared features of ASM mines, which we believe is a more useful approach. In light of this, we have included the following sentence in our introduction (L115-6):

*“Despite the term’s widespread usage no standard definition of ASM exists; aa practical approach may involve one or more of a group of shared features(12).”*

With regards to including the definition in the abstract, given the ambiguity of definition but widespread usage of the ASM term, and the limited number of words available in the abstract, we would respectfully request to not include such a discussion of the definition in the abstract. This would be in keeping with studies included in the article and the above mentioned ASM-focussed review(2). In addition to our addition to the introduction, we have also highlighted potential issues that result from this ambiguity in our discussion (see following point and response).

5. The prevalence of silicosis in stone mines of Rajasthan (India) is reported between 38-79% (<https://doi.org/10.1016/S2213-2600(15)00052-1>). The reported pooled prevalence is an underestimate. In India, small-scale mining is more prevalent and mainly in the unorganized sector.

Thank you for this helpful comment which, as well as ensuring the inclusion of population in the reference, has highlighted issues regarding the ambiguity of the ASM definition. This ambiguity has meant that, although populations under study may include miners who may meet a broad definition of ASM, they are not specifically referred to as such in the title, abstract, keywords or methods of their respective articles and therefore were not captured by the search criteria of our study. We have therefore included the following sentence in our limitations (L506-512):

*“Reflecting ambiguity in the definition of ASM, studies may include ASM but not specifically refer to them in their abstract or methods. For example, one study described “small scale” sandstone mines from Rajasthan, India in their introduction but did not meet our search criteria. The study reported a silicosis prevalence (ILO >1/0) of 275/526 (52%)(68), in keeping with other unpublished reports from the area, suggesting a prevalence at least as high as the studies in our review(69).”*

6. “Our modelling demonstrated decreases in cumulative RCS are associated with reductions in silicosis and tuberculosis, with greater reductions at higher mean exposures.” – some numbers here would be more informative. This sentence is an interpretation and not the results.

Thank you for this comment. With regards to the inclusion of numbers, as the primary aim of the modelling is descriptive (the “observation of disease risks in counter-factual scenarios”) we feel that a narrative summary best captures this aim, within the limited confines of an abstract. We would therefore respectfully request to keep this format. Within the results section and in the supplementary material, where we have less issues of competing space, we are able and do use figures to illustrate our results. With regards the second part of the comment, we have clarified to remove the implication of causation in this abstract sentence. It now reads (L75-77):

*“Our modelling demonstrated decreases in cumulative RCS are associated with reductions in silicosis and tuberculosis, with greater reductions at higher mean exposures.”*

Introduction
1. Silicosis also affects the TB treatment outcomes (<https://www.nature.com/articles/s41598-023-30012-4>). Authors might consider adding it.

Many thanks for this reference. As noted above, it’s omission was an oversight and thank you for brining it to our attention. We have included it both in the introduction (L101) and discussion (L493).

*“TB in those with silicosis is associated with poor outcomes(6,7).”*

2. Merge smaller paragraphs.

Thank you; we have merged paragraphs in response to this.

3. How do ASM and LSM differ? Please also define ASM first.

We have responded to this comment as above, noting the challenges in doing so.

4. Most of the Indian studies focus on ASM as mining is many a times in the informal sector, illegal, and unregulated. Reference to such Indian studies and context would be helpful.

We have chosen to do so in the introduction (L101) discussion (L494-5 and L506-512), many thanks for raising this issue.

Materials and methods
1. Well-written

Results
1. “Variables in the model are held constant, unless otherwise stated, at values of: a baseline silicosis prevalence of 2%, an increased odds of 1.3 of silicosis per 1 mg/m3-year increase in RCS exposure, a baseline TB incidence of 200 cases per 100,000 per year, an increased odds of 1.05 of TB per 1 mg/m3-year increase in RCS exposure, an increased odds of TB of 4x in those with silicosis, a randomly distributed baseline prevalence of HIV of 2% and an increased odds of TB of 4x in those with HIV.” – what was the basis of these assumptions?

These assumptions are based on a review of the literature which is outlined in Supplementary materials File 6. In certain cases, for example baseline TB incidence and HIV prevalence, they are necessarily contextual. In these cases the figures are based on appropriate estimates from included studies and are discussed in the Supplementary material.

2. Any results on – at what levels of silica dust does the risk of TB increase?

This is clearly a question of relevance from a public health perspective. Our modelling approach has assumed RCS as a continuous variable and silicosis presence as a binary variable. Poisson regression assumes an exponential relationship for continuous variables, such as RCS. Our choice of coefficient and approach to model RCS was based on the available literature (as outlined in Supplementary File 6). However, as discussed in our limitations with respect to RCS and silicosis, alternative strategies, for example a flexible approach to RCS, may provide a truer representation of the relationship although have not been performed in previous studies.. Furthermore, other factors – such as HIV prevalence which has been shown to combine multiplicatively with silicosis – mean that levels of RCS that lead to TB increases above baseline are likely context specific. Taken together, therefore, being able to accurately estimate at what levels of RCS TB risk increases above baseline is both context specific and would require an accuracy beyond which this modelling approach is able to provide.

Discussion
1. Well-written. Collaborative TB-silicosis activities can be discussed (<https://www.nature.com/articles/s41598-023-30012-4>).

Many thanks – it is included as noted above.

2. Need for maintaining data and addressing underreporting of silicosis should be highlighted.

3. Data from periodic medical examinations would also be a ‘mine’ to analyze, provided they are truly reported data.

Conclusion
1. Collaborative TB-silicosis activities can be proposed to be included in national programs.

With respect to the three comments above, we have amended our recommendations to address the recording and maintenance of routinely collected data. Although not specifically referred to in this section, this would be considered to included CXR screening. The importance of CXR screening has however been amended and highlighted in L492. We hope that the collaborative element of screening activities is adequately incorporated in recommendation 4 (L538-542).

3. *“We must fund and support national industrial hygiene programs that have specific remits to include ASM and record and maintain routinely collected data.*
4. *These programs should work with National TB programs in ASM communities, particularly to scale up TB preventative therapy and diagnostics.”*
