# Supplementary material for: Silicosis, tuberculosis and silica exposure among artisanal and small-scale miners: A systematic review and modelling paper
Source: PLOS Glob Public Health. 2023 Sep 21;3(9):e0002085. doi: 10.1371/journal.pgph.0002085 (PMC10513209; doi:10.1371/journal.pgph.0002085)
Supplement: Attachment — Submitted filename: Response to Reviewers.docx [file pgph.0002085.s012.docx]

Response to Reviewers

Journal requirements

1. Please review your reference list to ensure that it is complete and correct. If you have cited papers that have been retracted, please include the rationale for doing so in the manuscript text, or remove these references and replace them with relevant current references. Any changes to the reference list should be mentioned in the rebuttal letter that accompanies your revised manuscript. If you need to cite a retracted article, indicate the article’s retracted status in the References list and also include a citation and full reference for the retraction notice.

I have reviewed all referenced and I am happy they are correct. There are no retracted articles.

2. Please insert an Ethics Statement at the beginning of your Methods section, under a subheading 'Ethics Statement'. It must include:

1) The name(s) of the Institutional Review Board(s) or Ethics Committee(s)

2) The approval number(s), or a statement that approval was granted by the named board(s)

3) (for human participants/donors) - A statement that formal consent was obtained (must state whether verbal/written) OR the reason consent was not obtained (e.g. anonymity). NOTE: If child participants, the statement must declare that formal consent was obtained from the parent/guardian.

I have included an ‘Ethics Statement’ saying the following:

## Ethics statement

This review utilised only published data, as such no ethics clearance was required.

3. We noticed that you used "unpublished" in the manuscript. We do not allow these references, as the PLOS data access policy requires that all data be either published with the manuscript or made available in a publicly accessible database. Please amend the supplementary material to include the referenced data or remove the references.

I have removed the article which refers to unpublished data and replaced it with an article which uses publishes studies (L508).

Reviewer #1: No further comments. The authors have addressed my comments.

Many thanks – no changes made.

Reviewer #2: Thank you for making the changes and congratulations on writing a very important paper. Last few words:
1. You might want to correct the spelling on L483 from “challenging” to “challenges”.

Many thanks for pointing this out; I have done so.

2. A recent review has been published which highlights prevalence of silicosis and silico-tuberculosis in India (<https://occup-med.biomedcentral.com/articles/10.1186/s12995-023-00379-1>). The authors might want to go through it and the references which it cites for inclusion in the main text or analysis, if felt important. The high prevalence of silicosis and TB in the mining population as compared to other occupations can be considered to be highlighted.

Many thanks for this reference and for highlighting the papers within it. I believe the reviewed studies in this paper highlight the issue of definition within the informal and small industries. I have included it at L508 with the following: *“Reflecting ambiguity in the definition of ASM, studies may include ASM but not specifically refer to them in their abstract or methods. For example, one study described “small scale” sandstone mines from Rajasthan, India in their introduction but did not meet our search criteria. The study reported a silicosis prevalence (ILO ≥1/0) of 275/526 (52%)(68). A separate study of the sandstone miners from Rajasthan does not make any reference to “small scale” nor informal mining(69). Importantly however published reports from informal mining and other silica-exposed industries in India suggest a prevalence at least as high as the studies in our review in most cases(70).”*
